# Paintable Decellularized‐ECM Hydrogel for Preventing Cardiac Tissue Damage

**DOI:** 10.1002/advs.202307353

**Published:** 2024-03-19

**Authors:** Jaewoo Lee, Seul‐Gi Lee, Beom‐seok Kim, Shinhye Park, M. Nivedhitha Sundaram, Byung‐gee Kim, C‐Yoon Kim, Nathaniel S. Hwang

**Affiliations:** ^1^ School of Chemical and Biological Engineering, Institute of Chemical Processes Seoul National University Seoul 151–742 Republic of Korea; ^2^ Department of Stem Cell Biology School of Medicine Konkuk University Seoul 143–701 Republic of Korea; ^3^ Interdisciplinary Program in Bioengineering Seoul National University Seoul 151–742 Republic of Korea; ^4^ Research Division EGC Therapeutics Seoul 08790 Republic of Korea; ^5^ Institute of Molecular Biology and Genetics, Institute for Sustainable Development (ISD) Seoul National University Seoul 08826 Republic of Korea; ^6^ Bio‐MAX/N‐Bio Institute of BioEngineering Seoul National University Seoul 08826 Republic of Korea; ^7^ College of Veterinary Medicine Konkuk University Seoul 05029 Republic of Korea

**Keywords:** heart‐decellularized extracellular matrix, hydrogel, myocardial infarction, Painting, vascularization

## Abstract

The tissue‐specific heart decellularized extracellular matrix (hdECM) demonstrates a variety of therapeutic advantages, including fibrosis reduction and angiogenesis. Consequently, recent research for myocardial infarction (MI) therapy has utilized hdECM with various delivery techniques, such as injection or patch implantation. In this study, a novel approach for hdECM delivery using a wet adhesive paintable hydrogel is proposed. The hdECM‐containing paintable hydrogel (pdHA_t) is simply applied, with no theoretical limit to the size or shape, making it highly beneficial for scale‐up. Additionally, pdHA_t exhibits robust adhesion to the epicardium, with a minimal swelling ratio and sufficient adhesion strength for MI treatment when applied to the rat MI model. Moreover, the adhesiveness of pdHA_t can be easily washed off to prevent undesired adhesion with nearby organs, such as the rib cages and lungs, which can result in stenosis. During the 28 days of in vivo analysis, the pdHA_t not only facilitates functional regeneration by reducing ventricular wall thinning but also promotes neo‐vascularization in the MI region. In conclusion, the pdHA_t presents a promising strategy for MI treatment and cardiac tissue regeneration, offering the potential for improved patient outcomes and enhanced cardiac function post‐MI.

## Graphical abstract

The heart decellularized extracellular matrix (hdECM) is one well‐known factor for the myocardial infarction (MI) regeneration. Here, a novel hdECM delivery method using paintable hydrogel with no size or shape limitations on application is shown. It exhibits stable wet cardiac tissue adhesion that is easily regulated with a simple treatment. Moreover, it demonstrates angiogenesis and MI regeneration abilities upon in vivo implantation.

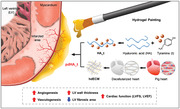

## 1 Introduction

The myocardial infarction (MI) is caused by a blockage in the coronary artery, which reduces blood supply to the heart muscle. This lack of oxygen leads to tissue necrosis and the loss of cardiomyocytes (CMs) due to a necrotic and apoptotic environment.^[^ ^1^, ^2^ ^]^ MI results in maladaptive ventricular remodeling, including scar tissue formation, ventricular wall thinning, and fibrosis, leading to impaired contractile function. As the heart is one of the least regenerative organs, additional therapeutic treatments are essential to encourage the regeneration of damaged cardiac tissue.^[^ ^3^, ^4^ ^]^

Current studies have demonstrated that tissue‐specific decellularized extracellular matrix (dECM) promotes tissue regeneration by mimicking the biophysical and biochemical composition of the tissue.^[^ ^5^, ^6^ ^]^ The structure and biochemistry of the dECM provide signals to cells that promote and regulate tissue growth, function, and repair.^[^ ^7^ ^]^ Furthermore, dECM promotes constructive remodeling and stimulates tissue formation at the implantation site.^[^ ^8^ ^]^ Due to these therapeutic effects, current studies have utilized the heart decellularized extracellular matrix (hdECM) for MI treatment. hdECM has been proven to be effective in reducing cardiac hypertrophy and fibrosis by regulating inflammation, apoptosis, and cardiac metabolism.^[^ ^9^, ^10^, ^11^ ^]^ Moreover, the hdECM has shown safety and potential therapeutic effects not only in small and large animal studies,^[^ ^12^, ^13^ ^]^ but also in Phase I clinical trials for post‐MI patients.^[^ ^14^ ^]^ It has also been shown that the hdECM containing angiogenic factors is sufficient to promote angiogenesis in the hdECM complex following in vivo transplantation.^[^ ^15^ ^]^ These results suggest that cardiac tissue‐specific hdECM may play a significant role in MI treatment by stimulating tissue remodeling and angiogenesis.^[^ ^16^, ^17^ ^]^

To deliver this versatile hdECM to the MI region, various methods have been employed, such as injections^[^ ^18^ ^]^ or patches.^[^ ^19^ ^]^ The hdECM was either injected with synthetic polymers using a syringe^[^ ^14^ ^]^ or injected invasively through a catheter.^[^ ^15^ ^]^ Patches made of synthetic polymers have been developed for the hdECM delivery.^[^ ^20^, ^21^ ^]^ However, the use of a syringe needle or sutures for injection or patch fixation may inadvertently cause additional injury to cardiac tissue.^[^ ^22^ ^]^ Utilizing adhesive patches can minimize additional tissue damage by eliminating the injections or the suturing process for patch fixation.^[^ ^22^, ^23^ ^]^ However, these adhesive patches also exhibited some limitations, including unexpected attachment to adjacent organs and fibrous adhesion. Thus, to avoid these phenomena, current studies attach the non‐adhesive layer to the adhesive patch.^[^ ^24^, ^25^ ^]^

**Scheme 1:**
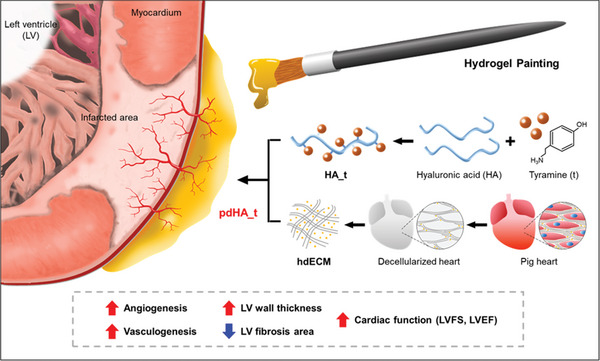
Paintable hdECM‐containing hydrogel for preventing cardiac tissue damage. Decellularized cardiac ECM was obtained by utilizing chemical decellularization method. hdECM‐containing tyramine conjugated hyaluronic acid was painted on the MI heart and showed stable wet adhesion by SA_Ty mediated oxidation process. The painted pdHA_t showed therapeutic effects, including preventing left ventricle (LV) wall thinning and angiogenesis.

This study proposes a new approach for delivering hdECM by using a paintable hdECM‐containing wet adhesive hydrogel, which minimizes additional tissue damage and undesirable adhesion by simple treatment. (**Scheme** **1**). The painting process can create a wet adhesive patch in situ, with no theoretical limit to size or shape, making it highly effective for scale‐up.^[^ ^26^ ^]^ To achieve this, catechol groups, known for their strong wet surface adhesiveness due to the reaction between oxidized catechol groups and other functional groups like thiols and amines, were utilized.^[^ ^27^, ^28^ ^]^ Recombinant tyrosinase from *Streptomyces avermitilis* (SA_Ty), an oxidizing agent, was used, which has previously shown high selectivity and reactivity to catechol‐based macromolecules.^[^ ^29^, ^30^ ^]^ We applied recombinant tyrosinase from the SA_Ty, which has previously been proven to exhibit high selectivity and reactivity to the catechol‐based macromolecules.^[^ ^31^, ^32^ ^]^ As a result, we demonstrated that pdHA_t could be easily painted with sufficient viscosity and exhibited controllable adhesion. Additionally, through the in vivo testing, we observed stable and immediate attachment of pdHA_t to the beating heart, along with therapeutic effects of hdECM, including prevention of ventricular wall thinning and promotion of angiogenesis.

## 2 Results

### 2.1 Optimization of Decellularizing Heart and Biochemical Analysis of hdECM

**Figure 1:**
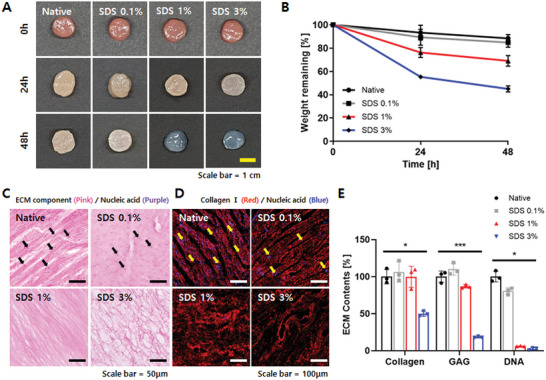
Decellularization and analysis of the porcine heart with varied SDS concentrations. A) Optical images of native and decellularized porcine heart tissues. B) ECM decomposition in the decellularization process at different SDS concentrations. C) H&E staining of native and decellularized porcine heart tissues. Arrows point to nuclei. Scale bar = 50 µm. D) Immunofluorescent staining for collagen I (red) and nucleic acid (blue) of native and decellularized porcine heart tissues. Arrows point to nuclei. Scale bar = 100 µm. E) Quantification of ECM components (GAG and collagen) and DNA at different SDS concentrations. Data are presented as mean ± SD. (n = 3, * ≤ 0.05, ***p ≤ 0.001).

The porcine heart was decellularized with different concentrations of sodium dodecyl sulfate (SDS) for 48 h. After 48 h, the decellularized heart was observed to be transparent when treated with both 1% and 3% SDS. Comparing the 1% and 3% SDS treatments, the overall size of the heart decreased in the 3% SDS group, indicating ECM degradation (**Figure** **1**A). The native (distilled water (DW) treated), 0.1% SDS, and 1% SDS groups showed slight degradation with 88.4 ± 3.3, 84.8 ± 4.10, and 69.1 ± 4.50% remaining weight, respectively. In contrast, the 3% SDS treated group degraded more than half of its initial weight, indicating massive ECM degradation (Figure 1B).

Hematoxylin & Eosin (H&E) staining and immunostaining with DAPI and collagen type 1 verified the absence of cells, cell debris, and maintenance of ECM. The native and 0.1% SDS groups showed a large number of cells in the ECM, but the 1% and 3% SDS‐treated groups showed no nucleic acid. Compared to the 1% SDS‐treated group, the 3% SDS‐treated group showed less dense ECM (Figure 1C,D). The hydroxyproline assay, dimethyl methylene blue (DMMB) assay, and PicoGreen assay were performed to quantify ECM components, such as collagen, glycosaminoglycan (GAG), and DNA, respectively (Figure 1E). The 3% SDS‐treated group has reduced ECM content due to ECM degradation. The DNA content in the 1% and 3% SDS groups was reduced by more than 90% compared to native cardiac tissue, resulting in a final concentration of less than 50 ng per mg ECM dry weight.

### 2.2 Fabrication and Characterization of the Paintable Hydrogel

**Figure 2:**
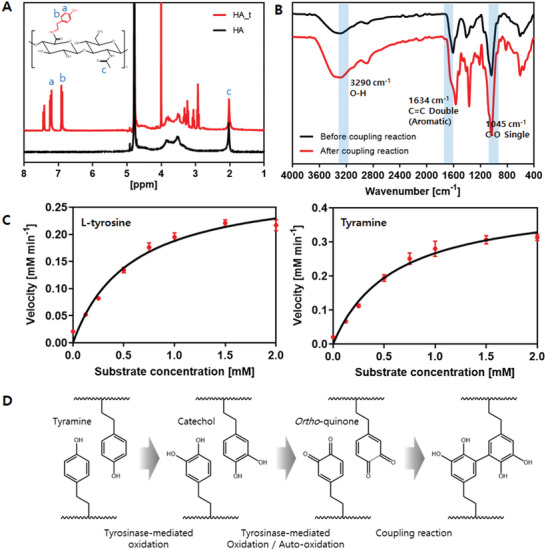
Synthesis and characterization of HA_t and SA_Ty. A) Detection of tyramine moiety of HA and HA_t using 300 MHz 1HNMR. B) Measurement of quinone formation and polymerization using FT‐IR analysis according to the tyramine coupling reaction. C) Measurement of SA_Ty specific activities to mono phenolic compound (L‐tyrosine and tyramine). D) HA_t crosslinking mechanism by tyrosinase‐mediated oxidation.

The HA–tyramine conjugate was prepared through carbodiimide chemistry, involving the Schiff base condensation and the formation of an amide bond between the carboxylic group of HA and the amine of tyramine. 1HNMR was performed to determine the degree of substitution of the tyramine‐conjugated hyaluronic acid (HA_t) (**Figure** **2**A). A three‐proton peak in the N‐acetyl group of HA appeared ≈2.0 ppm. Peaks of four aromatic protons in ortho‐ and meta‐ positions on the tyramine were detected at 6.8 and 7.2 ppm, respectively.^[^ ^33^ ^]^ The integrated areas of the aromatic protons' peak were 0.36 and 0.64, respectively, compared to the integrated area of the N‐acetyl peak. Based on the integrated areas, the rate of tyramine substitution was calculated to be 75.2%. FT‐IR analysis was further performed to determine the presence of the conjugated tyramine moiety. The broad band at 3290 cm^−1^ indicates the hydroxyl group of HA. Additional peaks at 1634 and 1045 cm^−1^ in HA_t correspond to the C═C and C─O bonds of the aromatic ring, respectively (Figure 2B).

The specific activity of SA_Ty for monophenolic substances (L‐tyrosine and tyramine) was assessed using the Beer‐Lambert law and the Michaelis‐Menten equation (Figure 2C).^[^ ^34^ ^]^ Kcat and Km values were calculated by measuring the reaction velocity of tyrosinase at various concentrations of monophenolic substrates (Figure S1, Supporting Information). The affinity to the substrate and the reaction rate of the generating product were represented by Km and Kcat, respectively. While SA_Ty showed a lower affinity for tyramine than L‐tyrosine, the enzyme reaction rate constants in both substrates were identical due to the faster reaction rate of tyramine. SA_Ty, a crosslinking agent with high reactivity, facilitates ortho‐quinone transformation before completing a coupling process initiated by tyrosinase‐mediated oxidation (Figure 2D).

### 2.3 Characterization of Paintable Hydrogel

**Figure 3:**
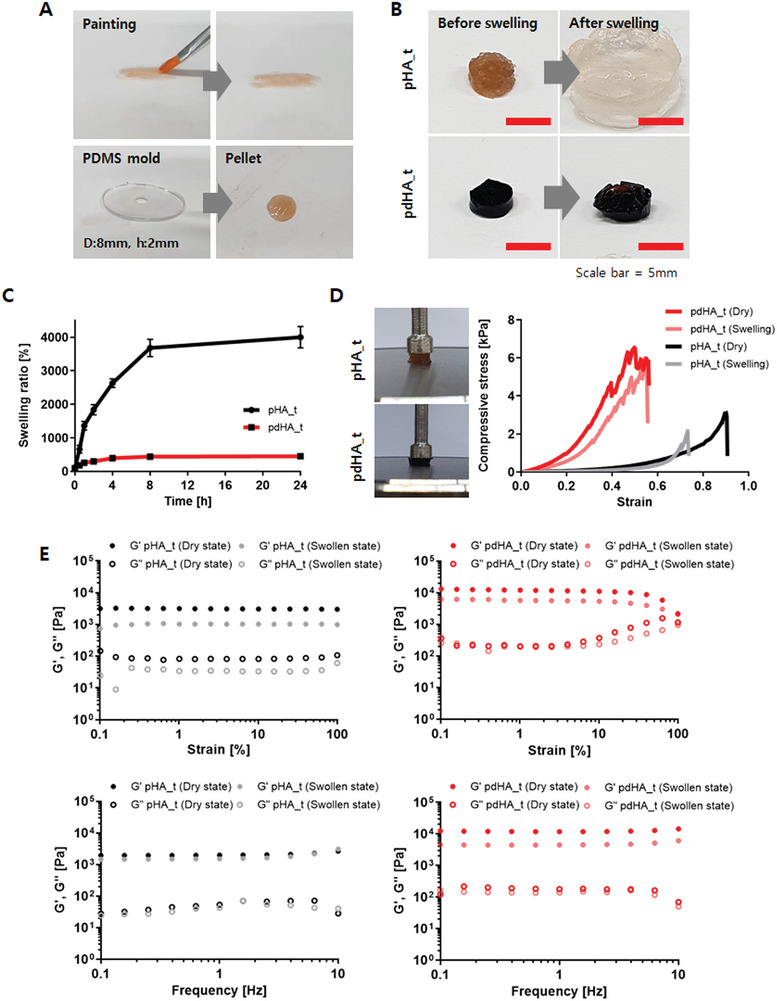
Characterization of paintable hydrogel with or without hdECM. A) early stage of the pdHA_t gelation after painting. B,C) Swelling behavior of fabricated hydrogel pellets, and measured swelling ratio after 24 h soaking in PBS. D) Compressive strength and E) rheological analysis (amplitude sweep and frequency sweep) before and after swelling paintable hydrogels.

A paintable hydrogel for cardiac repair should behave like a soft, flexible, and sticky paste with sufficient viscosity to be applied effectively on the heart. HA_t solution prepared with low concentrations, ranging from 1% to 3%, showed low viscosity and shrinkage, making it difficult to load the hydrogel on the commercial brush for painting. On the other hand, high concentration groups of 5% and 6% HA_t were hard to paint, as they did not form a coating with a uniform thickness (Figure S2A, Supporting Information). The hydrogel prepared with 4% HA_t, hdECM, and SA_Ty. The pdHA_t was easy to paint and formed a uniform and thin hydrogel layer on the substrate. Also, the prepared pdHA_t could be easily picked up with a commercial brush and filled a PDMS mold (**Figure** **3**A). Also, the painting method demonstrated the character of having no theoretical limitation in size or shape. In the scale‐up painting analysis, pdHA_t was successfully applied to the porcine heart with stable adhesion under various conditions, including water flush. Additionally, the pdHA_t can be painted in multiple layers on the porcine heart (Figure S3, Supporting Information). Rheological analysis was performed to determine the viscosity of pdHA_t compared with hydrogel without hdECM (pHA_t). In comparison to pHA_t, the viscosity of pdHA_t increased significantly, indicating that pdHA_t rapidly reached a sticky paste form (Figure S2B, Supporting Information).

The hydrogel prepared for application in the heart should exhibit minimal swelling to avoid cardiac tamponade.^[^ ^25^ ^]^ After 24 h of swelling in phosphate‐buffered saline (PBS), the size of pHA_t increased significantly compared to its initial size (Figure 3B). Furthermore, the swelling ratio, defined as the ratio of the original hydrogel weight to the swollen hydrogel weight, increased to 4000 ± 319.2% for pHA_t. In comparison, pdHA_t exhibited a swelling ratio of 452 ± 19.3% with only a minimal difference in its size (Figure 3C). Based on the swelling behavior of pHA_t and pdHA_t, mechanical characteristics of hydrogels before and after swelling were measured for further analysis. The compressive strength of hydrogel pellets with dimensions of 8 mm×2 mm (diameter × height) was measured. Although both pHA_t and pdHA_t showed a decrease in maximum compressive stress after swelling, pdHA_t demonstrated greater compressive strength than pHA_t before and after swelling (Figure 3D). Amplitude and frequency sweeps were performed to analyze the rheological behavior of the prepared hydrogels before and after swelling. In both amplitude and frequency sweeps, pdHA_t demonstrated a higher storage modulus (G’) of ≈12.04 ± 0.61 kPa and 12.36 ± 0.75 kPa in the dry state, and 5.78 ± 0.28 kPa and 4.73 ± 0.48 kPa in the swollen state, respectively. In contrast, pHA_t displayed significantly lower rheological behavior in both the dry and swollen states over the entire strain and frequency range (Figure 3E).

### 2.4 Adhesion Behaviors of the Paintable Hydrogel

**Figure 4:**
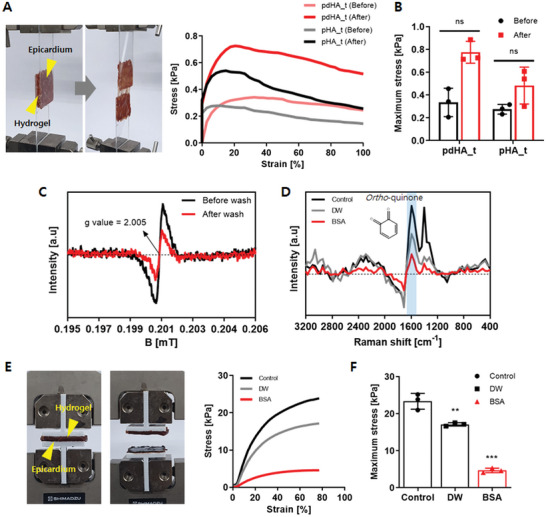
Adhesiveness and adhesion loss of paintable hydrogel. A) Measurement of adhesiveness by lap‐shear test and B) maximum stress of pdHA_t and pHA_t hydrogels before and after crosslinking. C) Measurement of existing radical before and after washing the paintable hydrogel. D) Quantification of residual quinone groups after washing with DW, and BSA solution. E) Adhesiveness measurement using tensile test and F) comparison of the maximum adhesive stress between groups. Data is presented as mean ± SD. (n = 3, ns indicates no significance, **≤0.01 and, ***≤0.001).

Previous research has shown that the catechol group has robust wet adhesion.^[^ ^29^ ^]^ Wet adhesion to the cardiac tissue was assessed using a standard lap‐shear test with porcine epicardium tissue. The pdHA_t showed a maximum adhesion stress of 0.33 ± 0.10 kPa and 0.78 ± 0.08 kPa before and after crosslinking. This indicates a significant difference in the adhesive stress after crosslinking pdHA_t. The pHA_t showed a maximum adhesion stress of 0.28 ± 0.03 kPa and 0.48 ± 0.13 kPa before and after crosslinking (**Figure** **4**A,B).

To prevent undesirable adhesion, we utilized two simple methods: eliminate free radicals that form quinone groups, and block quinone groups that trigger wet adhesion by interacting with amine or thiol groups on the tissue surface (Figure S4, Supporting Information). Electron Spin Resonance (ESR) demonstrated that washing the pdHA_t surface with DW decreased the quantity of free radicals by half compared to the unwashed surface (Figure 4C). The surface of pdHA_t was washed with solutions such as DW and BSA solution to block the existing quinones. Raman spectrometer analysis revealed a significant decrease in quinone intensity within the intense band at 1630 cm^−1^ when the BSA solution was used to wash the pdHA_t surface (Figure 4D).

To evaluate adhesion strength, a standard tensile test was conducted to assess the detachment strength of pdHA_t between the porcine epicardiums (Figure 4E). Compared to the control and DW‐treated pdHA_t, which showed maximum adhesion stress of 23.35 ± 1.75 kPa and 17.11 ± 0.37 kPa, the pdHA_t treated with the BSA solution showed a decreased maximum adhesion stress of 4.68 ± 0.51 kPa, respectively (Figure 4F).

### 2.5 Cytotoxicity of the Paintable Hydrogel

**Figure 5:**
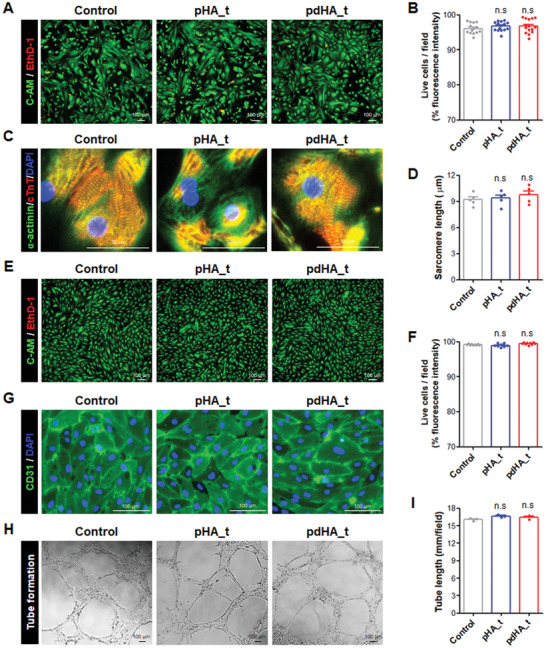
Cytotoxicity assessment of paintable hydrogel. A–D) Cytotoxicity assessment using hESC‐CMs following treatment of culture medium (Control) and eluated medium of pHA_t and pdHA_t incubation. A) LIVE/DEAD assay and B) quantification of live cells per field in hESC‐CMs between groups (Control: n = 14, pHA_t and pdHA_t: n = 15). Scale bar = 100 µm. C) Co‐staining for α‐actinin (green) and cTnT (red) and D) comparison of sarcomere length in hESC‐CMs between groups (n = 5). Scale bar = 50 µm. E–I) Cytotoxicity assessment using hESC‐ECs following treatment with each group. E) LIVE/DEAD assay and F) quantification of live cells per field in hESC‐ECs between groups (n = 9). Scale bar = 100 µm. G) Staining for CD31 in hESC‐ECs between groups. Scale bar = 100 µm. H) Tube formation and (I) comparison of tube length in hESC‐ECs between groups (n = 3). Scale bar = 100 µm. Data is presented as mean ± SEM. (ns indicates no significance; compared to control).

Prior to in vivo transplantation of the developed hydrogel, we evaluated the cytotoxicity of pHA_t and pdHA_t on the cells comprising the hear. CMs constituting the myocardium of the heart were prepared from human embryonic stem cells (hESCs; H9; Female) by induction following an established small molecules‐based differentiation protocol.^[^ ^35^ ^]^ The characterization of hESC‐CMs was verified by FACS (Over 90% expression of cTnT, a CMs‐specific marker), morphology (beating CMs), immunostaining (sarcomeric structure through co‐expression of cTnT and α‐actinin), and multi‐electrode array (MEA; Regular depolarization and repolarization) (Figure S5, Supporting Information). The hESC‐CMs were treated with culture medium (Control) and eluted medium (pHA_t and pdHA_t) for 24 h. LIVE/DEAD assay and immunostaining for CMs‐specific markers were performed. More than 96% of hESC‐CMs survived in all groups, and there were no significant differences between the groups (**Figures** **5**A,B). Additionally, all groups showed similar sarcomere lengths without inducing the collapse of sarcomeric structures (Figure 5C,D).

Endothelial cells (ECs) constituting the endothelium of blood vessels in and out of the heart were prepared by induction of hESCs following an established cytokines‐based differentiation protocol.^[^ ^36^ ^]^ The characterization of hESC‐ECs was verified by morphology (cobblestone‐shaped ECs), immunostaining (expression of CD31 and vWF, ECs‐specific markers), and Matrigel‐tube assay (tube forming ability, the angiogenic potential of ECs) (Figure S6, Supporting Information). After treating hESC‐ECs with the medium of each group (Control, pHA_t, and pdHA_t) for 24 h, LIVE/DEAD assay, immunostaining for CD31, and Matrigel‐tube assay were performed. More than 98% of hESC‐ECs survived in all groups without significant differences between the groups (Figure 5E,F), and the morphology of hESC‐ECs expressing CD31 was also similar (Figure 5G). In the study comparing the tube‐forming ability, which is a functional characteristic of ECs, all groups showed similar tube lengths without significant differences (Figure 5H,I).

### 2.6 Degradation Behavior of the Paintable Hydrogel

Degradation behavior was validated prior to long‐term in vivo implantation. In comparison to pHA_t, which degraded to 84.9 ± 8.7% after 72 h in PBS (pH 7.4), the pdHA_t showed no degradation. Following enzymatic degradation with hyaluronidase (100 U per sample dry weight mg), the weight loss of pdHA_t was substantially lower than that of pHA_t (Figure S7A, Supporting Information). Also, in the inflammatory conditions, pHA_t showed 72.3% and 64.5% of weight remaining in the collagenase type 1 and type 2 solution, respectively. In contrast, the weight of the pdHA_t slightly increased, which indicates that swelling occurs with negligible hydrogel degradation (Figure S7B, Supporting Information). Furthermore, to verify the in vivo degradation behavior, the weight of pHA_t and pdHA_t was measured at day 0, and the weight of the samples collected at 7, 14, 21, and 28 days after subcutaneous transplantation in rats was measured (Figure S7C, Supporting Information). Compared to the weight of the hydrogel before transplantation, on day 7, both groups showed a significant reduction in weight %, but there was no significant difference between the groups (degradation observed for pHA_t: 31.26 ± 14.02% vs pdHA_t: 32.62 ± 3.09%). Interestingly, after day 7, the weight of pHA_t (day 14: 48.24 ± 4.37%, day 21: 44.56 ± 8.68%, and day 28: 20.07 ± 10.05%) continuously decreased over time, while the weight of pdHA_t (day 14: 79.88 ± 1.30%, day 21: 89.80 ± 0.46%, and day 28: 93.07 ± 0.94%) gradually increased. At day 28, the weight of pdHA_t was found to be similar to the weight of pdHA_t observed before transplantation (Figure S7D, Supporting Information). As a result of performing H&E staining to check the image of the hydrogel over time, the completely crosslinked pdHA_t was compact on the inside at 7 and 28 days, and the hydrogel expanded further as day 28 progressed (Figure S7E, Supporting Information). On the other hand, the interior of pHA_t began to be degraded from day 7 along with the influx of many cells, and by day 28, the interior was so severely degraded that it lost its initial shape (Figure S7E, Supporting Information). Therefore, it was confirmed that the weight of pdHA_t gradually increased over time, while the weight of pHA_t decreased through extreme degradation.

### 2.7 Painting of Hydrogel on MI Model and Induction of Neovascularization

**Figure 6:**
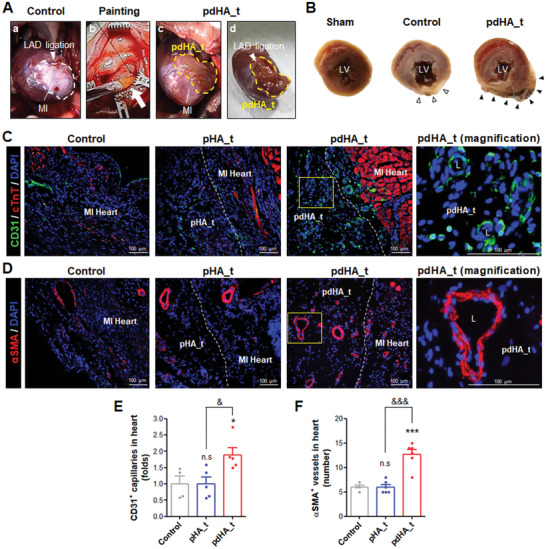
Validation of neovascularization by painting of paintable hydrogel on MI model. A) Heart images 28 days after MI induction and painting in Control or pdHA_t. MI area: white dotted line. pdHA_t: yellow dotted line. LAD ligation: white arrowhead. B) LV region according to transverse section of sham, Control, and pdHA_t. MI area: white arrowheads. pdHA_t: black arrowheads. C–F) Immunostaining of samples 28 days after MI induction and painting. C) Co‐staining for cTnT and CD31 at the lesion site between groups. L: lumen structure of vessels. Scale bar: 100 µm. D) Staining for αSMA at the lesion site between groups. Scale bar: 100 µm. E) Comparison of CD31^+^ capillaries density in the heart within the lesion site between groups (Control: n = 4, pHA_t and pdHA_t: n = 5). (F) Comparison of αSMA^+^ vessels number in the heart within the lesion site between groups (Control: n = 4, pHA_t and pdHA_t: n = 6). Data are presented as mean ± SEM. (* < 0.05 and *** < 0.001 (comparison with control); ns indicates no significance, ^&^ < 0.05, and ^&&&^ < 0.001).

After MI was induced through ligation of the left anterior descending artery (LAD), the untreated control group after 28 days showed clear white areas of the left ventricle, as nutrient and oxygen supply was cut off.^[^ ^37^ ^]^ To confirm whether the paintable hydrogel can actually show therapeutic effects in the MI model, the prepared samples (pHA_t and pdHA_t) were painted using a commercial brush on the LV after 5 mins of crosslinking time and allowed to harden for 3 mins with BSA solution treatment. When pdHA_t was painted on the LV and cross‐linked, it adhered well to the beating heart (**Figure** **6**A). As Figure 4B shows the difference in maximum adhesive stress between pHA_t and pdHA_t after crosslinking, pdHA_t was easier to paint, and it adhered well to wet LV areas than pHA_t (Data not shown). Additionally, we were able to successfully paint the hydrogel on the MI area of the beating heart. The painted hydrogel quickly crosslinked and adhered only to the site of application and did not exhibit any adhesion to the surrounding area despite the slow movement of the heart. This highlights the significance of using paintable hydrogel for treating MI compared to the conventional suturing technique (Figure S8, Supporting Information). This result shows that the paintable hydrogel prevents lesion adhesion that normally occurs after surgery and will also not restrict the movement of the beating heart.^[^ ^38^ ^]^ Degradation scoring (S1: fully degradation, S2: >50% degradation, and S3: non‐degradation) was performed to compare the degree of attachment of pHA_t and pdHA_t to the heart 28 days after MI induction and painting of hydrogel. Similar to the degradation behavior after subcutaneous transplantation shown in Figure S4 (Supporting Information), pdHA_t (S2: 12.5% and S3: 87.5%, n = 7/8) hardly degraded and was maintained at the site of application in the heart, unlike pHA_t (S1: 40%, S2: 40%, and S3: 20%) (Figure S7, Supporting Information). To examine in detail the lesions in the LV and the attachment of the hydrogel, the heart was transected. Unlike the morphology of sham, control showed a thin, white LV wall at the site of the lesion. In contrast, pdHA_t had fewer lesions, and the adhered paintable hydrogel was observed on the LV wall (Figure 6B).

There are two main processes: 1) angiogenesis, which mainly consists of capillaries dominated by ECs, 2) vasculogenesis, which mainly forms blood vessels in which smooth muscle cells (SMCs) surround ECs, such as arteries and veins.^[^ ^39^ ^]^ As a result of co‐staining for CD31 and cTnT, pdHA_t formed more CD31+ capillaries than Control and pHA_t in the MI heart area (the area where cTnT is sparsely or densely expressed) (Figure 6C,E). The CD31+ capillaries with lumen were also formed in the painted hydrogels and were significantly more distributed in pdHA_t than in pHA_t (Figure S10A, Supporting Information). Comparing vasculogenesis by staining α‐smooth muscle actin (αSMA; SMCs specific marker), pdHA_t formed significantly more αSMA+ vessels than Control and pHA_t in the MI Heart (Figure 6D,F) and painted hydrogel areas (Figure S10B, Supporting Information). Additionally, cTnT staining showed reduced myocardium with MI‐induced necrosis of CMs in control, unlike sham, but pdHA_t prevented severe myocardium disintegration. And, through this data, it was found that CMs do not move with paintable hydrogel. (Figure S11, Supporting Information). These results demonstrated that pdHA_t, which contains cardiac tissue‐specific decellularized ECM, generated more active angiogenesis and vasculogenesis than pHA_t.

### 2.8 Therapeutic Effects of Paintable Hydrogel on MI

**Figure 7:**
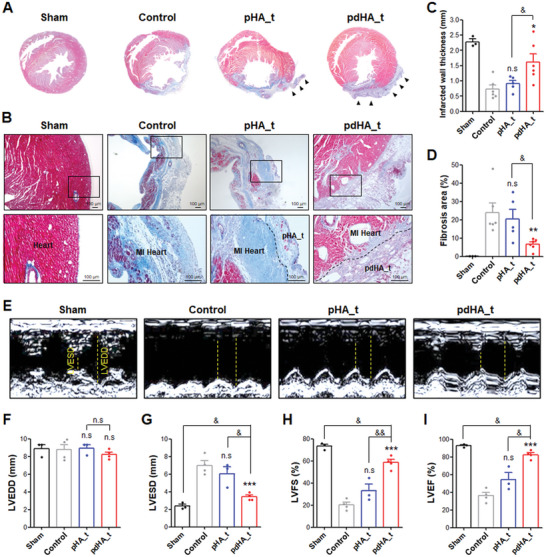
Analysis of therapeutic effects of paintable hydrogel on MI model. A‐D) MT staining of samples 28 days after MI induction and painting (Sham: n = 3, pHA_t: n = 5, Control and pdHA_t: n = 6). A) Comparison of LV between groups according to MT staining and verification of hydrogel adhesion. Paintable hydrogel: black arrowheads. B) Magnified image of the LV lesion area. Scale bar: 100 µm. Comparison of C) infarcted wall thickness and D) fibrosis area of lesion site between groups. E–I) Echocardiography measurement 30 days after MI induction and painting (Sham and pHA_t: n = 3, Control and pdHA_t: n = 4). E) Echocardiography images of LV between groups. Indication of LVESD and LVEDD with yellow dotted lines. Comparison of F) LVEDD, G) LVESD, H) LVFS, and I) LVEF between groups. Data are presented as mean ± SEM. (* < 0.05, ** < 0.01, and ***p < 0.001 (comparison with control); ns indicates no significance, ^&^ < 0.05, and ^&&^ < 0.01).

In addition to vascular collapse, morphological and pathological symptoms resulting from MI include thinning and severe fibrosis of the LV wall.^[^ ^34^ ^]^ Masson's trichrome (MT) staining was performed to compare sham, control, pHA_t, and pdHA_t groups. All three groups (control, pHA_t, and pdHA_t) exhibited thinner LV walls and blue‐stained fibrotic areas, indicating the presence of MI‐induced damage. Notably, in the pHA_t and pdHA_t samples, the painted hydrogel (arrowheads) was also stained blue, suggesting that the hydrogel adhered to the damaged area (**Figure** **7**A). Comparing the LV wall thickness and fibrosis area to the sham group, the control group showed significant thinning and fibrosis, confirming the successful induction of MI (Figure 7B–D). Based on this, the therapeutic effect of pHA_t and pdHA_t on the MI area was compared. The pdHA_t group showed a significantly thicker LV wall and less fibrosis than the control and pHA_t groups (Figure 7B–D). Furthermore, the importance of macrophages in MI treatment research is highlighted, as the balance between pro‐inflammatory (M1) and anti‐inflammatory (M2) macrophages in the infarct area plays an important role in infarct expansion and adverse cardiac remodeling.^[^ ^40^, ^41^ ^]^ As a result of co‐staining for CD86, an M1 specific marker, and CD206, an M2 specific marker, it was confirmed that pdHA_t, unlike control and pHA_t, significantly improved the distribution of CD206 cells while reducing the distribution of CD86 cells at the infarction site (Figure S12, Supporting Information).

To further assess the therapeutic potential, echocardiography was performed to evaluate cardiac function (Figure 7E). Left ventricular end‐systolic diameter (LVESD), left ventricular end‐diastolic diameter (LVEDD), left ventricular fractional shortening (LVFS), and left ventricular ejection fraction (LVEF) were measured as the main parameters.^[^ ^42^ ^]^ There were no significant differences in LVEDD among all groups (Figure 7F), but LVESD, LVFS, and LVEF showed significant differences between the groups (Figure 7G–I). The control group exhibited a significant decrease in LVFS and LVEF, and an increase in LVESD compared to the sham group. Comparing the pHA_t and pdHA_t groups, the increase in LVESD due to MI was significantly reduced in the pdHA_t treated group (Figure 7G). Moreover, LVFS and LVEF, which decreased significantly due to MI, were significantly increased in the pdHA_t group (Figure 7H,I).

## 3 Discussion

In this study, wet tissue adhesive paintable hydrogel was developed for delivering cardiac tissue‐specific hdECM for MI regeneration. For the therapeutic benefits, the hdECM has been demonstrated as a therapeutic material for stimulating cardiac tissue remodeling and angiogenesis by mimicking native cardiac tissue. For the therapeutic benefits without acute rejection following implantation, the hdECM should minimize cellular components while maintaining native ECM components by optimized decellularization method, which is largely dependent on source tissue characteristics such as species and size.^[^ ^43^, ^44^, ^45^ ^]^ Through the optimized chemical decellularization method, obtained hdECM showed maintained ECM components, including collagen and GAGs, and decreased cellular components with a final concentration of less than 50 ng per mg ECM dry weight which is a safe level to prevent immunological reactions.^[^ ^21^ ^]^ A paintable hydrogel was selected as a novel approach for hdECM delivery was prepared with the proper concentration of HA_t as soft and sticky paste behavior in sufficient hydrogel viscosity. Moreover, Catechol‐conjugated HA_t, in conjunction with SA_Ty, as an oxidant, formed a tightly linked hydrogel and showed stable and firm attachment to the wet epicardium after being painted. Additionally, in terms of rheology, phHA_t exhibited a larger storage modulus than the loss modulus in both amplitude and frequency sweet tests, indicating that the pdHA_t was stable and behaved viscoelastically in hydrogel form. Through the hdECM delivery analysis, the inclusion of hdECM in the pdHA_t gave multiple beneficial effects compared with pHA_t, including rapid increase in the hydrogel viscosity, decreased swelling ratio with minimal loss in compressive strength, and improved mechanical and rheological properties.

Recently, studies to develop a patch as a strategy for infarcted cardiac tissue regeneration have been published.^[^ ^46^, ^47^, ^48^ ^]^ However, the absence of adhesive characteristics of these patches requires further treatment, such as sutures or light irradiation, for a stable attachment, and these additional treatments cause secondary damage to the cardiac tissue.^[^ ^49^ ^]^ The pdHA_t showed strong adhesive strength by catechol‐based interaction and it can minimize secondary tissue damage. Although strong and stable wet adhesion of the pdHA_t was beneficial to long‐term in vivo analysis, undesirable adherence with adjacent organs or tissues, such as the rib cage and lung, and fibrous adhesion were also expected and posed serious issues.^[^ ^50^ ^]^ Previous studies attached an additional layer on the adhesive patch for an anti‐adhesion barrier.^[^ ^24^, ^31^ ^]^ Unlike previous studies, we prevented undesirable adhesion by simply washing with BSA solution, which can eliminate free radicals and block the quinone groups, and it demonstrated that using a catechol group can easily manage its adhesiveness. In addition to these versatile behaviors of the pdHA_t, low cytotoxicity and low biodegradability of the pdHA_t were also suitable for long‐term MI treatment. After 28 days of in vivo implantation, it was demonstrated that the pdHA_t can restore serious heart damages (e.g., ventricular wall thinning and fibrosis) that continues to worsen after infarction occurs and can retain the cardiac pump performance by assisting the LV wall laxity. Moreover, in severe ischemic diseases such as MI, it is difficult to recover with only short‐term spontaneous angiogenesis, resulting in myocardial necrosis.^[^ ^34^, ^51^ ^]^ Therefore, attempts have been reported to transplant patches containing MSCs and cardiac stem cells (CSCs)‐derived exosomes with pro‐angiogenic effects.^[^ ^12^ ^]^ In this study, even if exogenous cell extracts were not included, pdHA_t showed promotion of angiogenesis/vasculogenesis during in vivo transplantation since hdECM possesses abundant collagen, fibronectin, and laminin components suitable for the functioning of cardiovascular constituting cells.^[^ ^52^, ^53^, ^54^ ^]^ This study suggested a new approach to deliver hdECM by wet adhesion paintable hydrogel. Also, it is expected that pdHA_t can be utilized in various ischemic disease treatments with its manageable adhesiveness and therapeutic ability, including neo‐vascularization.

## 4 Conclusion

MI is an acute cardiac disease that causes fibrosis, ventricular wall thinning, and heart malfunction. The main objective of this article is the synthesis of a paintable hydrogel containing the hdECM for the treatment of MI. The optimization of the decellularization method for porcine heart tissue resulted in the absence of cellular components while maintaining the native components of the ECM. The fabrication of a paintable hydrogel using catechol‐modified HA_t, hdECM, and a crosslinking agent (SA_Ty) proved its suitability for cardiac repair. The pdHA_t hydrogel exhibited a paintable nature with appropriate viscosity, and minimal swelling while maintaining desirable mechanical and rheological properties. The pdHA_t hydrogel also demonstrated strong wet adhesion to the cardiac tissue after crosslinking which offers an advantage over conventional suturing techniques. Washing the hydrogel with BSA solution effectively prevented undesired adhesion to neighboring organs by eliminating free radicals and blocking quinone groups. The cytotoxicity evaluation showed the biocompatible nature of the pdHA_t hydrogel with CMs and endothelial cells. Also, the degradation profile of the hydrogel indicated its stability in physiological conditions. In vivo analysis showed the therapeutic effect of the pdHA_t in a MI model. The pdHA_t hydrogel was successfully painted and adhered well to the wet LV of the heart with minimal degradation over 28 days. Compared to the control group and the group treated with pHA_t, the pdHA_t hydrogel significantly reduced the thinning of the LV wall and the formation of fibrotic areas. It promoted angiogenesis and vascularization, as evidenced by the increased formation of CD31+ capillaries and αSMA+ vessels in the MI heart area. Furthermore, pdHA_t treatment improved cardiac function parameters, including LVESD, LVFS, and LVEF, compared to the control and pHA_t groups. The therapeutic effects observed in this study suggest that the pdHA_t could effectively restore heart damage caused by MI. The paintable hydrogel with tissue‐specific decellularized material represents a valuable addition to the field of cardiac tissue engineering and regenerative medicine, offering new possibilities for improving patient outcomes and enhancing cardiac function post‐MI.

## 5 Experimental Section

### Materials

As synthetic biomaterials, Hyaluronic acid (Molecular weight = 1000–1,200 kDa, NutriVita, USA) was used. Tyramine hydrochloride, pepsin, and Triton X‐100 were purchased from Sigma‐Aldrich (Seoul, Korea). The 4‐(4,6‐Dimethoxy‐1,3,5‐triazine‐2‐yl)−4‐methylmorpholinium Chloride (DMTMM) was purchased from Tokyo Chemical Industry Co., Ltd (TCI, Tokyo, Japan), Porcine Heart was purchased from Biozoa (Seoul, Korea), SDS was purchased from Bio‐Rad (California, USA).

### Porcine Heart Decellularization

The porcine heart was chopped into 3 mm thick slices and rinsed in cold water for 24 h. The porcine heart was then decellularized by treating it with 0.1, 1, and 3% SDS for 48 h, followed by 1% Triton X‐100 treatment for 6 h. The weight remaining (%) was measured with respect to initial weight of the porcine heart. Treatment with Triton X‐100 was done to efficiently remove the remaining SDS. The decellularized heart was sterilized for 4 h using 0.1% peracetic acid in 4% ethanol, rinsed with sterilized DW for 48 h and was then lyophilized. The lyophilized decellularized heart was then digested with pepsin (decellularized heart:pepsin = 10:1, weight ratio) in 0.5 M acetic acid. The digested solution was neutralized to physiological pH (pH 7.0) and was lyophilized. The lyophilized hdECM was pulverized with a blender to obtain the hdECM powder that was used for further studies.

### hdECM Biochemical analysis

Native and decellularized porcine hearts were fixed, embedded in paraffin, and sectioned using a cryotome (Leica RM2125 RTS, Germany) for histological analysis. Sectioned tissues were stained with H&E and immunofluorescence staining for collagen type I and nucleic acid was performed. Biochemical assays were performed to quantify residual ECM components (collagen and GAG) and DNA for assessing the degree of decellularization. Residual collagen was quantified by the hydroxyproline assay, GAG was quantified using the 1,9‐DMMB assay, as described in the previous studies^[^ ^21^ ^]^ and DNA was quantified using the Quant‐iT PicoGreen dsDNA Assay Kit (Thermo Fisher Scientific) according to the manufacturer protocol. Absorbance or fluorescence was measured using spectrophotometer (TECAN, Switzerland).

### HA_t Synthesis and Characterization

Briefly, HA (1 g) was dissolved in pH 5.5 PBS buffer (100 mL). DMTMM was reacted for 1 hour at 70 °C (molar ratio: HA:DMTMM = 1:2). Tyramine hydrochloride was added (molar ratio: HA:tyramine = 1:4) and was reacted for 24 h. HA_tyr solution so formed was dialyzed for 48 h and was then lyophilized. The degree of substitution of the tyramine was measured by ^1^HNMR (AVANCE III HD 300 MHz). FT‐IR spectra was obtained at wavelengths from 400 to 4000 cm^−1^ using an FT‐IR spectrometer (Bruker TENSOR27, Germany).

### SA_Ty Synthesis and Characterization

The Sa‐Ty was synthesized and characterized following protocol published by our group in our previous studies.^[^ ^55^ ^]^ Tyrosinase *E.coli* was inoculated with ampicillin in autoclaved LB medium and grown overnight in a shaking incubator at 37 °C. The cultured *E.coli* was then transferred to fresh LB medium and cultured at 37 °C for 3 h. When the OD600 value of the bacteria solution was between 0.6 and 0.8, 1 M isopropyl β‐D‐1‐thiogalactopyranoside (IPTG) and 1 M CuSO_4_*5H_2_O were added to induce protein expression, and the cultured medium was kept in a shaking incubator at 18 °C for 20 h. Cell pellets were then collected by ultra‐centrifugation (4000 rpm and 10 min) and washed twice with 50 mM tris‐HCl buffer (pH 8.0) (5 mL). The cells were lysed at 4 °C for 20 min using an ultra‐sonicator (VC505, USA) and was then centrifuged at 4 °C for 30 min. The expressed enzymes were purified by His‐tag purification with Ni‐NTA agarose bead. The obtained SA_Ty was filtered using a 0.22 µm syringe filter, collected in a 10 kDa filter tube and mixed with autoclaved 75% glycerol solution. The concentration of purified enzymes was calculated by Bradford assay. After adding pH 8.0 tris buffer, 10 nM CuSO_4_, tyrosinase, and 2 mM L‐tyrosine, SA_Ty was incubated at 37 °C for 30 min and absorbance at 475 nm was measured. The specific activity of SA_ty was calculated using the Beer‐Lambert law and Michaelis‐Menten equation. The activity of tyrosinase with different concentrations of L‐tyrosine and tyramine at 37 °C was measured using UV‐spectroscopy (475 nm).

### Fabrication of Paintable Hydrogel

Prepared HA_t was dissolved at 4% (w/v) in DW and mixed with 1% (w/v) of hdECM to make the pdHA_t. In contrast, pHA_t contained only HA_t solution without addition of hdECM powder. Both pdHA_t and pHA_t was crosslinked with 1% (v/v) of SA_Ty solution. Crosslinked pdHA_t and pHA_t hydrogels were used for further studies.

### Viscosity of Paintable Hydrogel

Viscosity of pHA_t and pdHA_t hydrogels were measured in the Demo lab (Anton‐Paar Korea). After adding SA_Ty to the HA_t solution (W and W/O hdECM), shear rate of 1 1/s was applied on the hydrogel for 1 h and the corresponding viscosity measurement was obtained.

### Swelling Behavior of Paintable Hydrogel

The pHA_t and pdHA_t hydrogels were molded on hole (d:8 mm and h:2 mm) in the PDMS mold. The fabricated hydrogel pellets were then dipped in PBS (10 mL) and the weight was measured for 24 h. The swelling ratio was calculated by using the following equation, swelling ratio = (Ws‐Wi)/Wi, where Wi and Ws are the initial weight and swollen hydrogel weight, respectively.

### Mechanical Properties of Paintable Hydrogel

Before and after swelling, hydrogel pellets were cut into similar sizes using an 8 mm biopsy punch. The pellets were pulled to failure with 5 mm min^−1^ speed during which the load and displacement were recorded using a universal tensile machine (UTM‐ Shimazu, EZ‐SX STD, Japan).

### Rheological Behavior of Paintable Hydrogel

Dynamic rheological measurements were carried out by performing amplitude and frequency sweep analysis before and after swelling hydrogel pellets. Rheological analysis was evaluated in the Demo lab (Anton‐Paar Korea) using Rheometer (MCR 302, Measuring cell: P‐PTD & H‐PTD 200, Measuring System: PP 25, Anton‐Paar, Austria). Amplitude sweeps measured storage modulus G’ and loss modulus G″ as shear strain (%) increased from 0.1 to 100 at a fixed frequency of 1 Hz. Frequency sweeps measured the storage modulus G’ and loss modulus G’ while increasing the frequency from 0.1 to 10 Hz with 1% fixed strain.

### Adhesion Behavior of Paintable Hydrogel

pdHA_t and pHA_t hydrogels were applied on epicardium with adhesion area of width 2.5 cm and length 2.5 cm in both tensile and lap‐shear tests. Both adhesion tests were performed with UTM (Shimazu, EZ‐SX STD, Japan) modules by following the ASTM standards for tensile (ASTM F2258) and lap‐shear (ASTM F2255) tests, respectively. For analysis of loss in adhesion upon washing the hydrogels, the hydrogel applied on the epicardium was washed with DW and BSA solution and then test was performed. In the tensile test, the hydrogel applied on epicardium without washing served as the control.

### Electron Spin Resonance (ESR) Analysis

For residual radical quantification, pdHA_t hydrogel pellet (d: 4 mm and h: 1 mm) was fabricated using PDMS mold. The residual radical present before and after washing the hydrogel pellet was measured. ESR measurement was performed in the National Center for Inter‐university Research Facilities (NCIRF) using EMXplus‐9.5/12/P/L system (Bruker, Germany).

### Raman Spectrometer Analysis

After washing hydrogel pellets with DW and BSA solution, quinone quantities of non‐treated hydrogel pellet (control) and washed hydrogel pellet was measured by RAMAN spectrometer (DXR2xi, Thermo Fisher Scientific, USA) and the Raman shift was obtained from 400 to 3200 cm^−1^.

### Cell Culture

hESC (H9; WiCell; Madison, WI, USA; Information: female and Catalog#WA09) were prepared for differentiation into CMs and ECs. The isolation of hESCs was performed following the procedures approved by Institutional Review Board (IRB) at the Konkuk University Medical Center of Korea (approval no. KUH1280080). Undifferentiated hESCs were cultured on Matrigel (Corning Inc.; Corning, NY, USA)‐coated culture dish with StemMACS iPS‐Brew XF medium (Miltenyi Biotec Korea; Gangnam, Seoul, Korea).

### Differentiation of hESC Derived CMs

To initiate differentiation into hESC derived CMs (hESC‐CMs), hESCs were seeded on Matrigel‐coated dishes using maintenance culture medium supplemented with 10 µ_M_ Y‐27632 (Tocris Bioscience, Bristol, UK). After that, the medium was changed every day for 3 days until the confluency reached 90%. At day 0, cells were treated with 6 µM CHIR99021 (Tocris) in RPMI1640 + B27 supplement without insulin (B27‐ medium; Thermo Fisher Scientific; Waltham, MA, USA). After 2 days, the medium was changed to B27‐ medium supplemented with 2 µ_M_ C‐59 (Selleckchem; Houston, TX, USA) for another 48 h. At day 4, the medium was replaced with Advanced MEM (Thermo Fisher Scientific) with 1% GlutaMax (Thermo Fisher Scientific) and 1% Penicillin/Streptomycin (Thermo Fisher Scientific), and freshly changed every 48 h until beating occurs. When beating cells appeared on day 10, cells were detached using TrypeLE (Thermo Fisher Scientific) and subculture on new Matrigel‐coated dishes. From day 12 to 18, the medium was replaced with glucose‐free RPMI1640 + B27 supplement (B27+) containing 4 mM sodium L‐lactate (Sigma‐Aldrich; St. Louis, MO, USA) to obtain pure hPSC‐CMs only. After purification, the medium was replaced with Advanced MEM every 2 days for up to 30 days, and hESC‐CMs were cultured to mature.

### Differentiation of hESC Derived ECs

For differentiation into hESC‐derived ECs (hESC‐ECs), low density hESCs were seeded onto Matrigel‐coated dishes using maintenance culture medium supplemented with 10 µ_M_ Y‐27632. After that, the medium was changed every day for 3 days until the confluency reached 20–30%. At day 0, cells were treated with 6 µ_M_ CHIR99021 in B27‐ medium for 48 h. Then, Endothelial growth medium‐2 (EGM‐2; Lonza; Gampel, Gampel‐Bratsch, Switzerland) containing VEGF (50 ng mL^−1^), BMP4 (20 ng mL^−1^), and bFGF (20 ng mL^−1^) was replaced daily for 3 days. At day 5, differentiated cells were detached with 0.05% Trypsin/EDTA and suspended in EGM‐2 containing 1:100 APC‐conjugated human CD31 antibody (Thermo Fisher Scientific). After 20 min, CD31+ cells (hESC‐ECs) were sorted by fluorescence‐activated cell sorting (FACS), and the sorted hESC‐ECs were cultured in 0.1% gelatin‐coated dishes and subculture was performed.

### FACS Analysis

Generated hPSC‐CMs and hPSC‐ECs were fixed in 4% paraformaldehyde (PFA; Biosesang; Seongnam, Gyeonggi, Korea) for 20 min and washed with PBS. Then, hESC‐CMs were stained with 1:100 PE‐conjugated human anti‐cardiac troponin T antibody (cTnT; Thermo Fisher Scientific) diluted in FACS solution at 4 °C for 30 min, and hESC‐ECs were stained with 1:100 APC‐conjugated human CD31 antibody. Stained cells were analyzed using FACS Calibur and Cell Quest software (BD Biosciences, San Jose, CA, USA) after washing.

### Multi‐Electrode Array (MEA)

In order to verify whether changes in electrophysiological signals of hESC‐CMs appeared in the medium containing the eluate of pHA_t and pdHA_t, analysis was performed using MEA. First, a 24‐well MEA plate was coated with fibronectin (50 µg mL^−1^) at the center of the well containing the electrodes for 1 h. The coated fibronectin was removed and medium containing 60 000 cells (6 µL) was seeded in drop form. After 2 h, it was confirmed that the CMs were attached to the electrodes, and medium (500 µL) was added. The medium was changed every 48 h until field potential (FP), an electrophysiological signal appearing in CMs, was observed. After ≈7 days, the MEA plate was directly transferred from the incubator to the MEA device Maestro Edge version (Axion BioSystems Inc, Atlanta, GA, USA). Environmental controls (37 °C and 5% CO2) were used to maintain temperature and pH. When FP appeared, it was recorded for 5 min and the plate was removed. After culturing the CMs of the recorded MEA plate for 24 h in a medium containing the eluate of pHA_t and pdHA_t, the change in FP was measured again in the MEA device and recorded for 5 min. Raw data recorded in AxIS software were analyzed using Axis Metric Plotting Tool (Axion BioSystems Inc)

### Eluate of Paintable Hydrogel

To verify the cytotoxicity of pHA_t and pdHA_t, pHA_t and pdHA_t were painted on a culture dish, respectively, and cell culture medium (Advanced MEM for CMs culture and EGM‐2 for ECs culture) was added to the dish. One day later, the eluate was harvested and centrifuged at 3 200 rpm, 15 min, 4 °C to secure the supernatant excluding debris.

### Live/Dead Assay

A LIVE/DEAD Viability/Cytotoxicity Kit (Invitrogen; Waltham, Massachusetts, USA) was used to identify the viability of the cells in the medium containing the eluate of pdHA_t and pdHA_t. The working solution was prepared by diluting 2 µ_M_ Calcein AM (C‐AM; stock volume: 4 mM) and 4 µ_M_ Ethidium homodimer‐1 (EthD‐1; stock volume: 2 mM) in PBS with 2% FBS. After washing the cells cultured in the eluted medium once with PBS, a working solution was added and incubated for 30 min at 37 °C incubator. The C‐AM was detected at the FITC (green) wavelength and EthD‐1 at the TRITC (red) wavelength. All images were analyzed using a fluorescence microscope, fluorescence intensity was measured using Image J, and the percentages of C‐AM and EthD‐1 to the sum of fluorescence intensities were compared.

### Tube Formation

Tube formation was used to identify the functionality of hESC‐ECs in the medium containing the eluate of pHA_t and pdHA_t. Cold Matrigel (300 µl) was put into a 24‐well plate and solidified for 30 min at RT. Eluate with 7 × 10^4^ hESC‐ECs was added on the solidified Matrigel. After 12 h, formed tubes were photographed using microscope, and quantified for tube length using image J.

### Immunofluorescence staining (IF)

The cultured hESC‐CMs and hESC‐ECs were fixed with 4% PFA for characterization. In addition, fixed heart samples, 28 days after MI induction and painting, were soaked in graded sucrose (15% and 30%) for 48 h and then embedded in OCT compound. The cryosections were sliced at a thickness of 9 µm using a cryostat set at −20 °C. For staining the cultured cells and cryosections of heart, the samples were incubated with a blocking solution (PBS containing 0.03% Triton‐X100 and 3% normal goat serum) for 30 min at RT. Subsequently, the sections and cells were incubated in the primary antibodies diluted in the blocking solution for overnight at 4 °C. Primary antibodies used: anti‐α‐actinin (1:200; Sigma‐Aldrich; A7811), anti‐cardiac troponin T (cTnT; 1:200; abcam; ab45932), anti‐CD31 (1:200; abcam; ab9498 and ab28364), anti‐α‐smooth muscle actin (αSMA; 1:200; abcam; ab5694), anti‐Vimentin (1:200; abcam; ab8978), anti‐CD86 (1:200; abcam; ab220188), and anti‐CD206 (1:200; abcam; ab64693). After overnight incubation, they were washed three times with PBS and incubated in secondary antibodies diluted in blocking solution for 2 h at RT. The secondary antibodies like: Alexa 488 goat anti‐mouse IgG (1:1000; Invitrogen; A11001) and Alexa 594 goat anti‐rabbit IgG (1:1000; Invitrogen; A11012) were used. The samples were stained with DAPI (1:1000; Thermo Fisher Scientific) for identification of nucleus. For cryosection, the slides were mounted using VECTASHIELD Mounting medium (Vector lab). All images were analyzed using fluorescence (Nikon TE2000‐U Japan) and confocal microscope. Quantification of CD31+ capillaries and αSMA+ vessels was performed using Image J software.

### Degradation Behavior in vivo of Paintable Hydrogels

Samples of pHA_t and pdHA_t that reached swelling equilibrium within 24 h were weighed before transplantation. This study was approved by the Animal Care and Use Committee of Konkuk University (IACUC NO.). All ethical codes applicable to animal experimentation and research were followed. Sprague Dawley (SD) rats (6 weeks old, male; ORIENTBIO INC.; Seongnam, Gyeonggi, Korea) were prepared. After the skin was incised, three samples of each group were implanted subcutaneously at regular intervals, and then sutured with 5‐0 silk (Ethicon; Somerville, NJ, USA). Samples were taken 7, 14, 21, and 28 days after transplantation, and tissue was removed as much as possible. The weight of the hydrogel samples from which tissue was removed was measured and compared with the weight before transplantation.

### MI Modeling in Rats and Painting of pHA_t and pdHA_t

This study was approved by the Animal Care and Use Committee of Konkuk University (IACUC NO. KU22019). All ethical codes applicable to animal experimentation and research were followed. SD rats (6 weeks old, male; ORIENTBIO INC.) were prepared. Prior to thoracotomy, rats were anesthetized with 2.5% inhaled isoflurane and an 18‐gauge intravenous catheter was intubated through the trachea. At the same time, rats were mechanically ventilated with medical grade oxygen. After adequate anesthesia for ≈10 min, a left intercostal thoracotomy was performed. Next, the ribs were opened using a retractor, and the pericardium was removed to secure the surgical site. MI was induced by ligating the LAD artery in the heart with a 6‐0 silk (Ethicon) suture. When it was confirmed that the left ventricular area (LV) had turned white following MI induction, the group without any treatment after surgery was set as control group. Next, the prepared samples (pHA_t and pdHA_t) were painted using a brush on the LV after 5 min of crosslinking time and allowed to harden for 3 min with BSA solution treatment. The total time required for the experiment was performed within 40–50 min. For comparison with the experimental group, Sham (normal group without surgery), was prepared along with other groups during the experimental period.

### H&E Staining

All rats were euthanized after 28 days of MI model creation, and the harvested hearts were fixed with 4% PFA. Fixed samples were embedded in paraffin after processing the tissues. Then, 5 µm‐sized sections were prepared using an HM 340E microtome (Thermo Fisher Scientific). H&E staining was performed to confirm the degradation of hydrogel.

### MT Staining

All rats were euthanized after 28 days of MI model creation, and the harvested hearts were fixed with 4% PFA. Fixed samples were embedded in paraffin after processing the tissues. Then, 5 µm‐sized sections were prepared using an HM 340E microtome (Thermo Fisher Scientific). MT staining was performed to determine the area of fibrosis. Paraffin sections from each group were deparaffinized and fixed overnight at RT in Bouin's solution. The fixed sections were stained with Weigert's iron hematoxylin solution for 10 min and with Biebrich Scarlet‐acid Fuchsin solution for 15 min at RT. Finally, sections were stained with Aniline Blue for 5 min. Sections were continuously washed between each staining step. The red color indicates CMs that survived MI, and the blue color indicates collagen fiber formation due to fibrosis. The percentage of area that showed fibrosis to the total left ventricular wall area was quantified using ImageJ software.

### Echocardiography

Echocardiography was performed to evaluate the damage due to MI modeling and the functional improvement of the heart after painting of pdHA_t. Echocardiography was performed 30 days after MI modeling. After anesthesia with isoflurane, physiological data on left ventricular systolic function were recorded using an echocardiography system (GE Vivid 7). The LVEDD and the LVESD were measured as main parameters, and the LVFS and the LVEF were calculated by the following equations.^[^ ^1^ ^]^

(1) $$\mathrm{LVFS}\left(\%\right)=\left[\left(\mathrm{LVEDD}-\mathrm{LVESD}\right)\mathrm{LVED}D^{-1}\right]\times 100$$

(2) $$\mathrm{LVEF}\left(\%\right)=\left[\left(\mathrm{LVED}D^{2}-\mathrm{LVES}D^{2}\right)\mathrm{LVED}D^{-2}\right]\times 100$$

### Statistical Analysis

All experiments were performed at least three times. Statistical analyses were performed using the GraphPad Prism software (La Jolla, CA, USA; Version 5). Data was presented as mean ± SEM or mean ± SD, and the statistical significance of the experimental results was calculated using one‐way ANOVA, Two‐way ANOVA, and t‐test. A value of *p* < 0.05 was considered statistically significant.

## Conflict of Interest

The authors declare no conflict of interest.

## Supporting information

Supporting Information

## Data Availability

The data that support the findings of this study are available from the corresponding author upon reasonable request.
