# Supplementary material for: Paintable Decellularized‐ECM Hydrogel for Preventing Cardiac Tissue Damage
Source: Adv Sci (Weinh). 2024 Mar 19;11(21):2307353. doi: 10.1002/advs.202307353 (PMC11151011; doi:10.1002/advs.202307353)
Supplement: Supplementary file 1 — Supporting Information [file ADVS-11-2307353-s001.pdf]

ADVANCED SCIENCE
Open Access

Supporting Information

for *Adv. Sci.*, DOI 10.1002/advs.202307353

Paintable Decellularized-ECM Hydrogel for Preventing Cardiac Tissue Damage

Jaewoo Lee, Seul-Gi Lee, Beom-seok Kim, Shinhye Park, M. Nivedhitha Sundaram, Byung-gee Kim, C-Yoon Kim and Nathaniel S. Hwang**

Supplementary Information

Paintable Decellularized-ECM Hydrogel for Preventing Cardiac Tissue Damage

Jaewoo Lee[1, &], Seul-Gi Lee[2, &], Beom-seok Kim[1], Byung-gee Kim, Shinhye Park[2], C-Yoon Kim[3, *], Nathaniel S. Hwang[1, *]

[1]School of Chemical and Biological Engineering, Seoul National University, Seoul, 151-742, Republic of Korea

[2]Department of Stem Cell Biology, School of Medicine, Konkuk University, Seoul, 143-701, Republic of Korea

[3]College of Veterinary Medicine, Konkuk University, Seoul, 05029, Republic of Korea

[&] These authors contributed equally to this work as first authors.

* These authors contributed equally to this work as corresponding authors.

*** Corresponding Author**

: Nathaniel S. Hwang, PhD

School of Chemical and Biological Engineering, Seoul National University, Seoul, 151-742, Republic of Korea.; E-mail: nshwang@snu.ac.kr,

: C-Yoon Kim, PhD

College of Veterinary Medicine, Konkuk University, Seoul, 05029, Republic of Korea.; E-mail: vivavet@konkuk.ac.kr

	L-Tyrosine, Monophenolase activity			Tyramine, Monophenolase activity		
	K_{cat} [S^{-1}]	K_m [μM]	K_{cat}/K_m [$M^{-1}S^{-1}$]	K_{cat} [S^{-1}]	K_m [μM]	K_{cat}/K_m [$M^{-1}S^{-1}$]
SA_Ty	0.61±0.005	524.62±23.05	0.12×10^4	1.00±0.06	780.11±23.90	0.12×10^4

Figure S1. Kinetic parameters of SA_Ty for substrate L-tyrosine and tyramine. Catalytic efficiency factors were obtained through the reaction between **s**ynthesized SA_Ty and the monophenol compounds. K_m, K_{cat}, and K_{cat}/K_m represent the substrate affinity, the reaction rate of the generating product, and the catalytic reaction rate constant, respectively. K_m and K_{cat} were obtained using the Michaelis-Menten equation. (n=3)

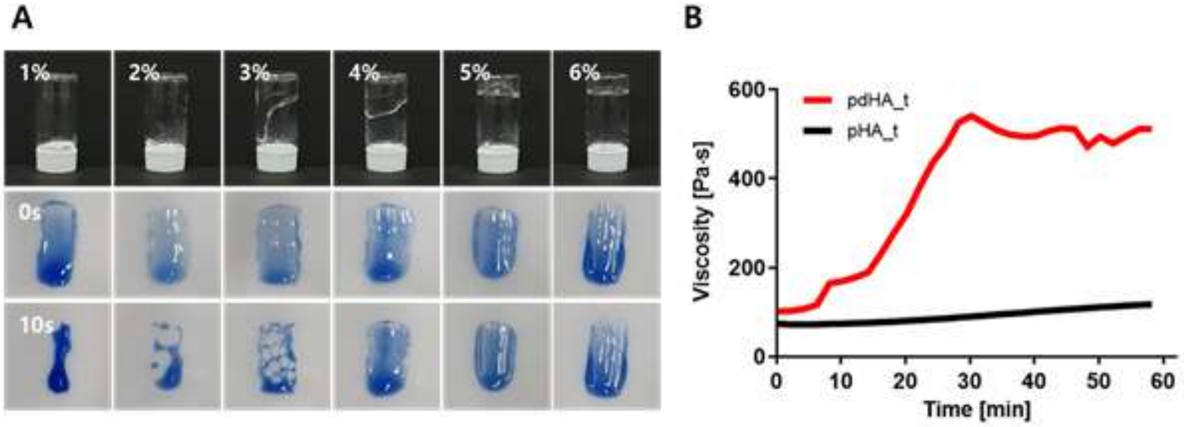

Figure S2. Comparison of viscosity for the paintable hydrogel. Sufficient viscosity is required for paintable hydrogel to be applied in a uniform thickness layer. A) Viscosity and painting behavior according to the concentration of the HA_t. B) Viscosity of paintable hydrogel according to the gelation process.

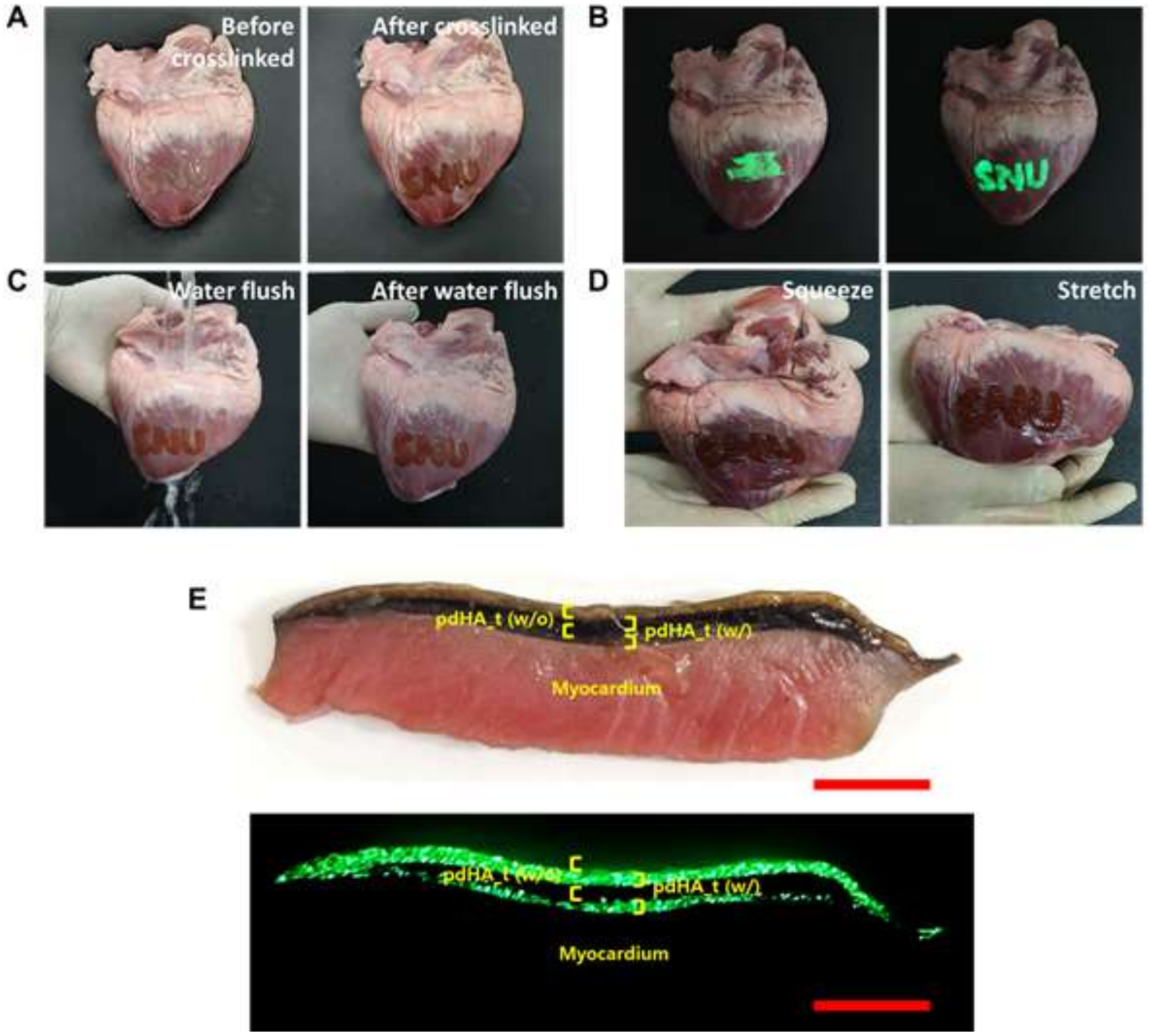

Figure S3. A scale-up painting analysis of the pdHA_t. A) Before and after crosslinking of painted pdHA_t, which had no shape changes. B) Painting the pdHA_t without shape (Left) and with shape (Right) on the porcine heart. C) There was no destruction in the painted pdHA_t after water flush, which indicates stable adhesiveness of the pdHA_t on the porcine epicardium. D) Squeeze and stretch cycles, which simply mimic the heart beating, also determined the stable and robust adhesion of the pdHA_t on the porcine epicardium. E) Multi-layered painting on the porcine epicardium.

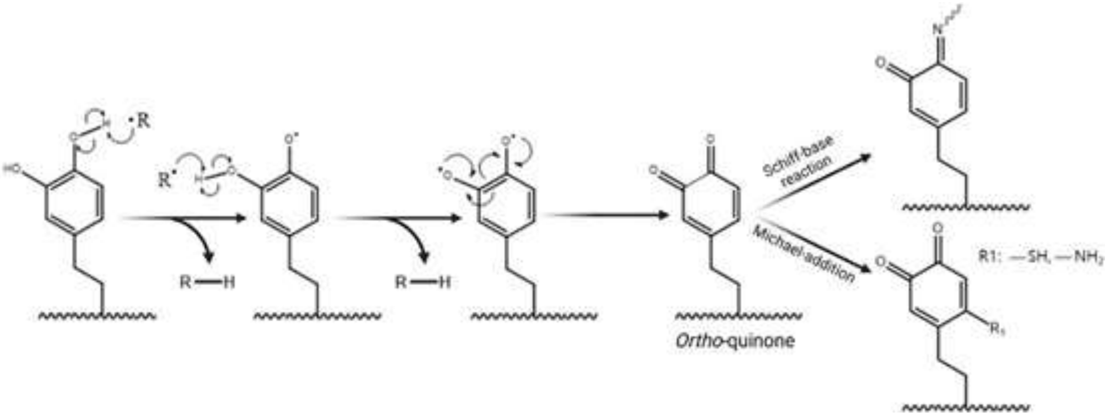

Figure S4. Adhesion mechanism of catechol group. Wet adhesion of catechol groups occurs in two stages. 1) *ortho*-quinone formation via tyrosinase-mediated oxidation, and 2) reaction of *ortho*-quinone with thiol or amine groups on the tissue surface.

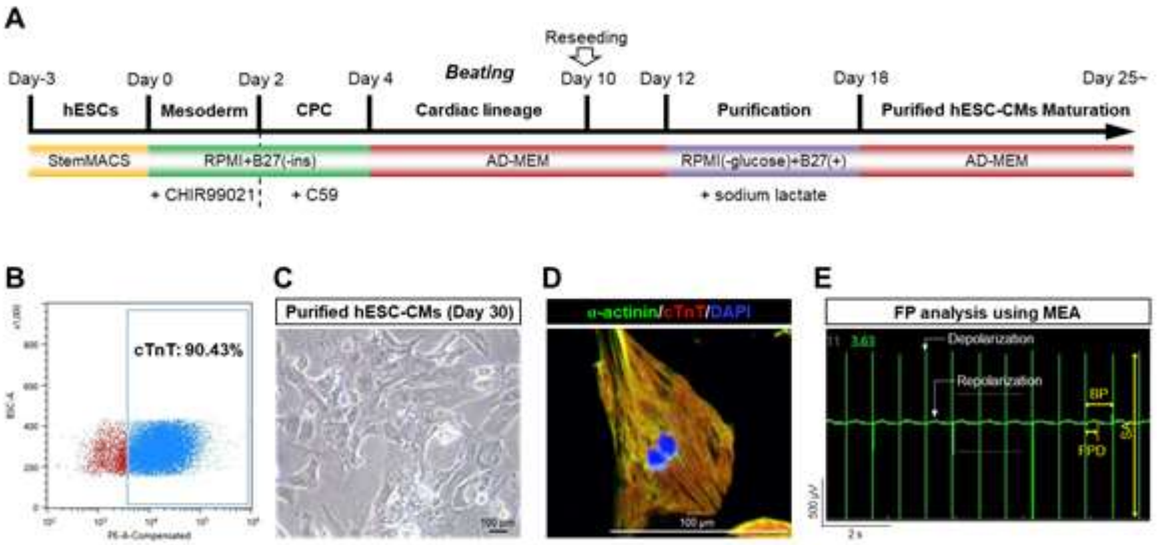

Figure S5. Characterization of hESC-CMs. A) Differentiation protocol based on small molecules to produce hESC-CMs. B) FACS analysis of cTnT to confirm the purity of hESC-CMs. C) Morphology of purified hESC-CMs at day 30 after differentiation. Scale bar: 100 μm. D) Co-staining for cTnT (red) and α-actinin (green) in

hESC-CMs. Scale bar: 100 μm. E) Evaluation of electrophysiological signals of hESC-CMs through MEA-based FP analysis. FPD: field potential duration. BP: beat period. SA: spike amplitude.

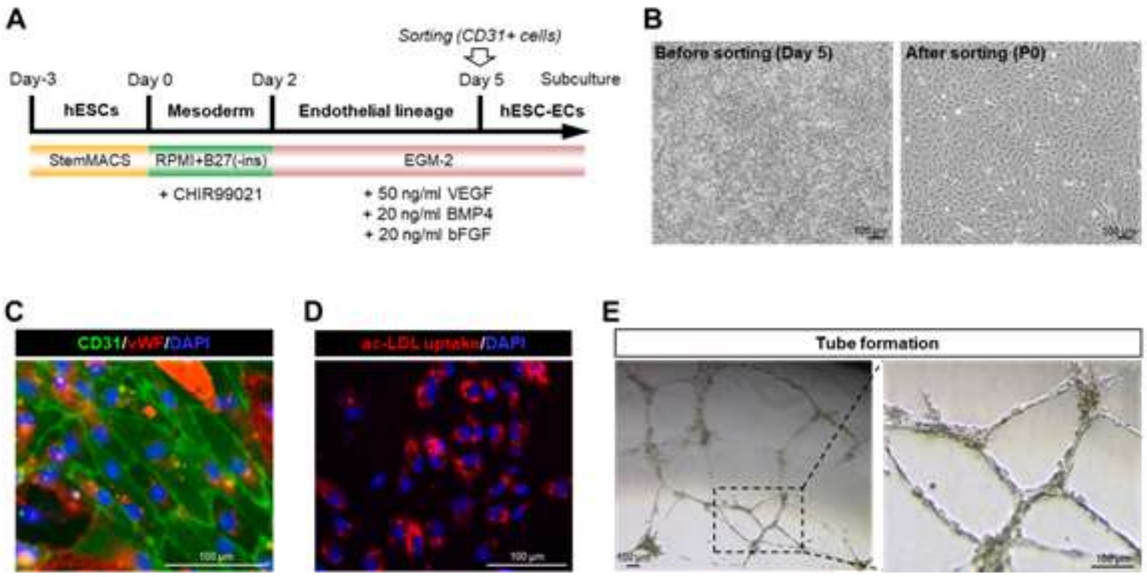

Figure S6. Characterization of hESC-ECs. A) Differentiation protocol based on cytokines to produce hESC-ECs. B) Morphology of hESC-ECs before (day 5) and after (P0) sorting. Scale bar: 100 μm. C) Co-staining for vWF (red) and CD31 (green) in hESC-ECs. Scale bar: 100 μm. D) Verification of ac-LDL uptake in hESC-ECs. Scale bar: 100 μm. (E) Tube formation assay of hESC-ECs to confirm angiogenic potential. Scale bar: 100 μm.

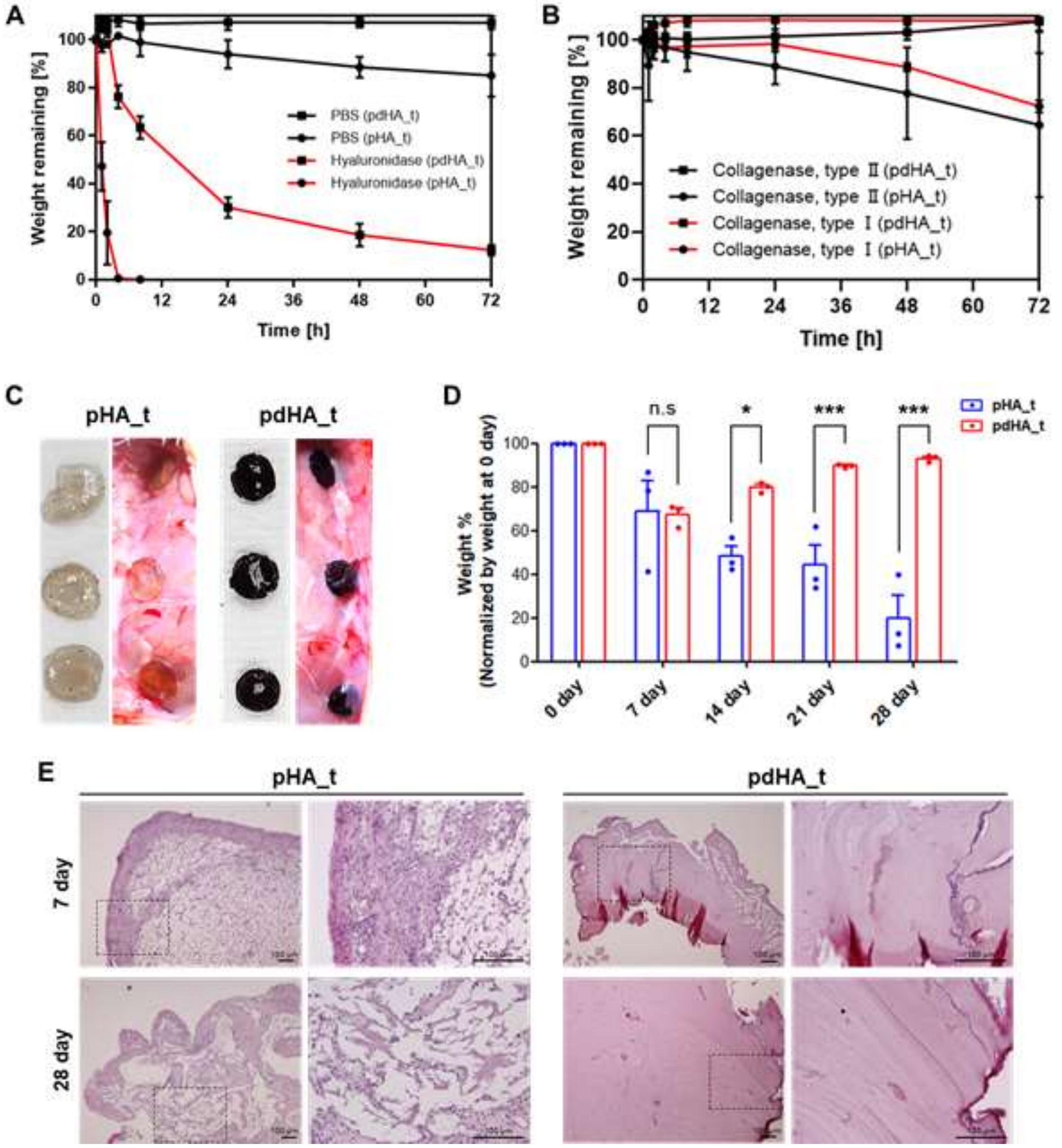

Figure S7. Verification of *in vitro* and *in vivo* degradation behavior of pHA_t and pdHA_t. A) Weight retention test following PBS and hyaluronidase treatment on pHA_t and pdHA_t. B) Weight retention test following collagenase type 1 and type 2 treatment on the pHA_t and the pdHA_t. C) Images of pHA_t and pdHA_t before and after subcutaneous transplantation (n=3). D) Weight % of hydrogel extracted on 7, 14, 21, and 28 days after implantation normalized with weight at day 0. E) H&E images of extracted hydrogel at 7 and 28 days to confirm degradation. Scale bar = 100 μm. Data are presented as mean ± SEM. (ns indicates no significance, *$p < 0.05$, and ***$p < 0.001$).

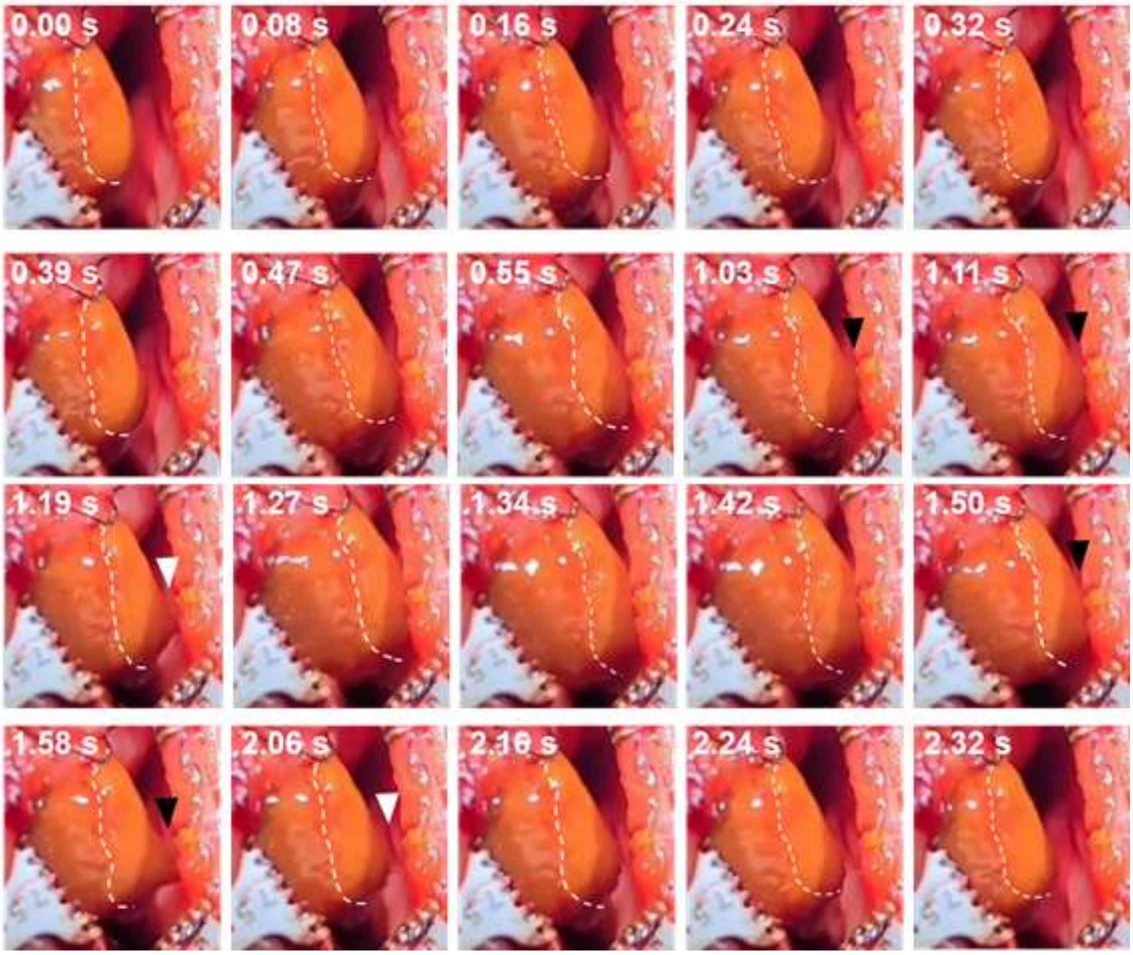

Figure S8. Verification of adhesion potential of pdHA_t in areas other than the heart. Snapshot of 0.25x slow motion of heartbeat video, after painting and crosslinking of pdHA_t on the heart. Sequential arrangement of images at intervals of 0.07-0.08 s. White dotted line: painted pdHA_t. Black arrow head: Timing when it hits the ribs. White arrow head: Timing when not attached anywhere other than the heart.

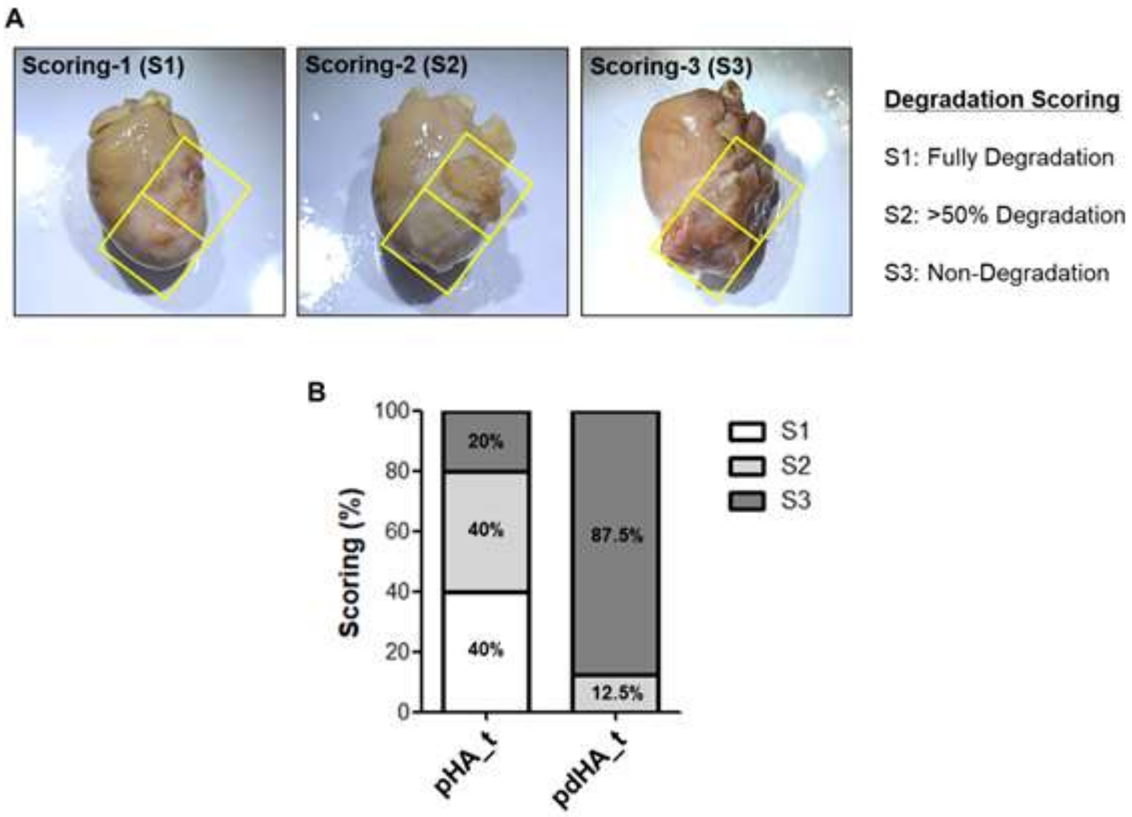

Figure S9. Percentage scoring indicating the in vivo degradation of pHA_t and pdHA_t applied on the heart. A) Images of painted hydrogel corresponding to each scoring from hearts harvested 28 days after MI induction and painting. B) Comparison of scoring (%) of pHA_t and pdHA_t (pHA_t: n=5 and pdHA_t: n=8).

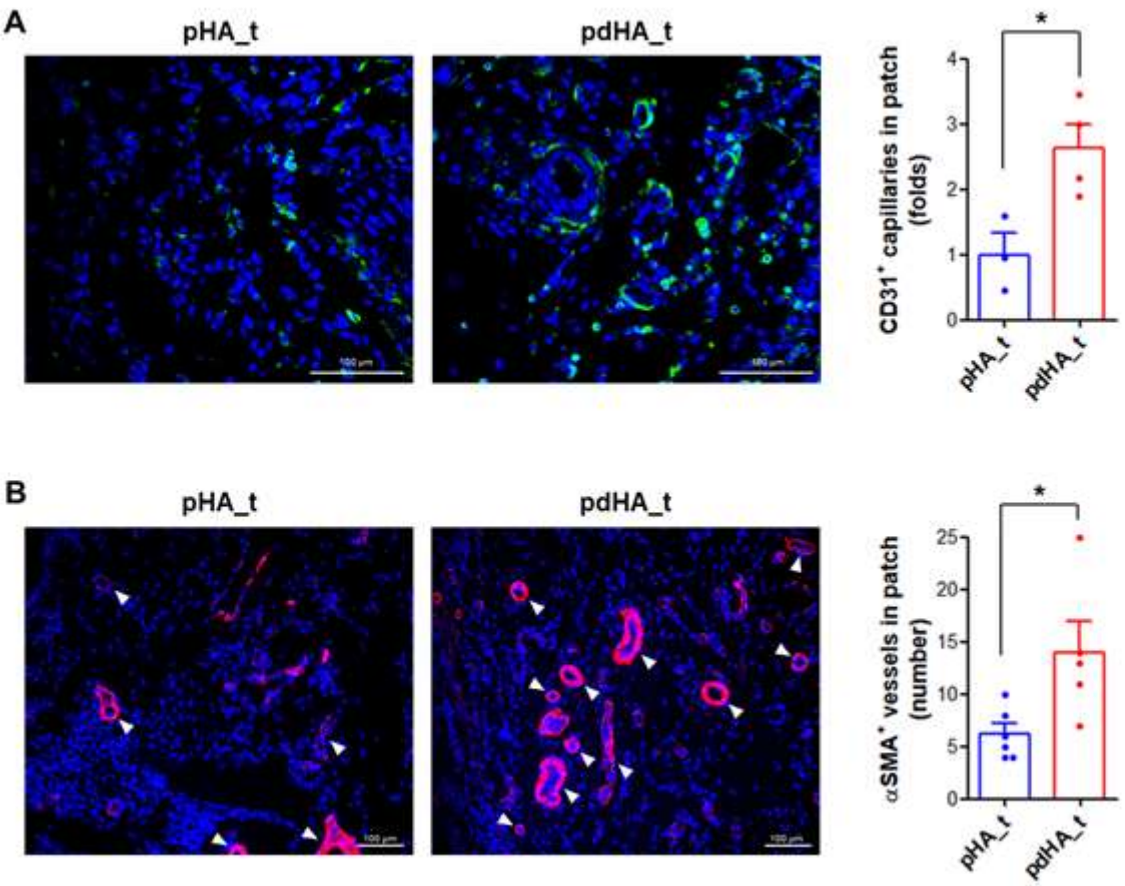

Figure S10. Comparison of angiogenic/vasculogenic potential in the painted hydrogel between pHA_t and pdHA_t groups. A) Staining for CD31 and comparison of CD31^{+} capillaries density at the painted hydrogel between groups (pHA_t: n=3 and pdHA_t: n=4). Scale bar: 100 μm. B) Staining for αSMA and comparison of αSMA^{+} vessels (white arrowheads) number at the painted hydrogel between groups (pHA_t: n=6 and pdHA_t: n=5). Scale bar: 100 μm. Data are presented as mean ± SEM. *p* value: $^{*}p < 0.05$.

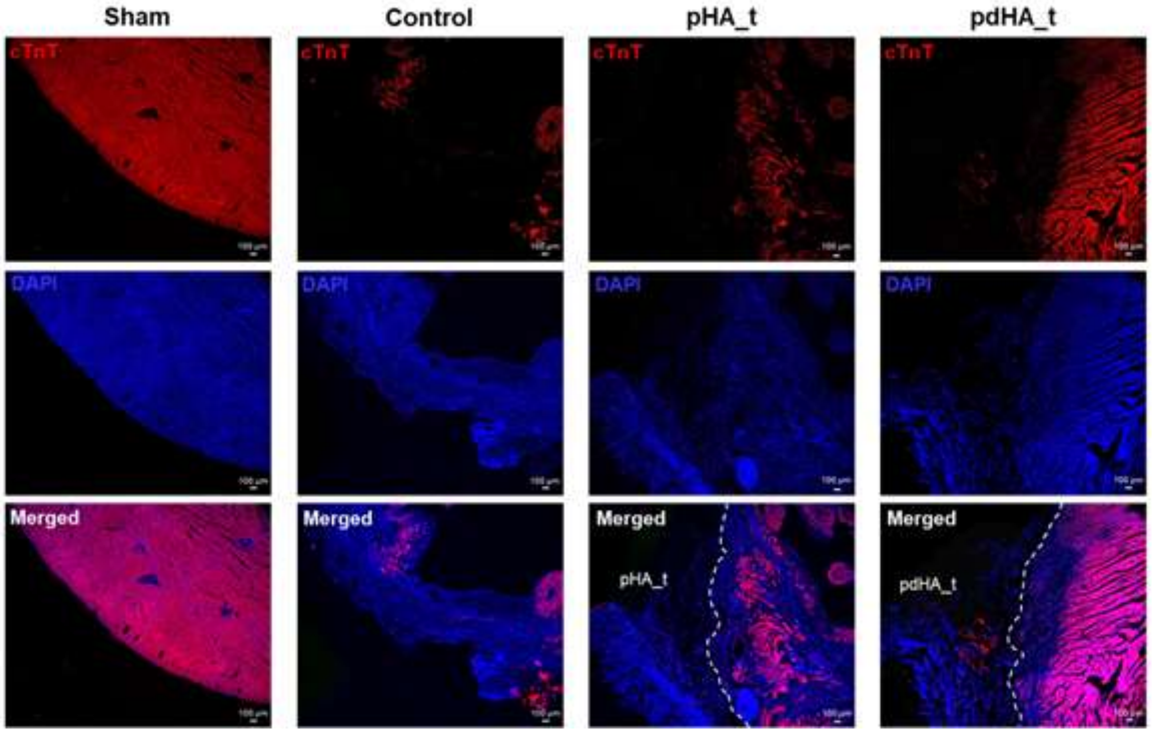

Figure S11. Comparison of the distribution of myocardium between groups. Immunostaining for cTnT in groups (Sham, Control, pHA_t, and pdHA_t) 28 days after MI induction and painting. Scale bar: 100 μm.

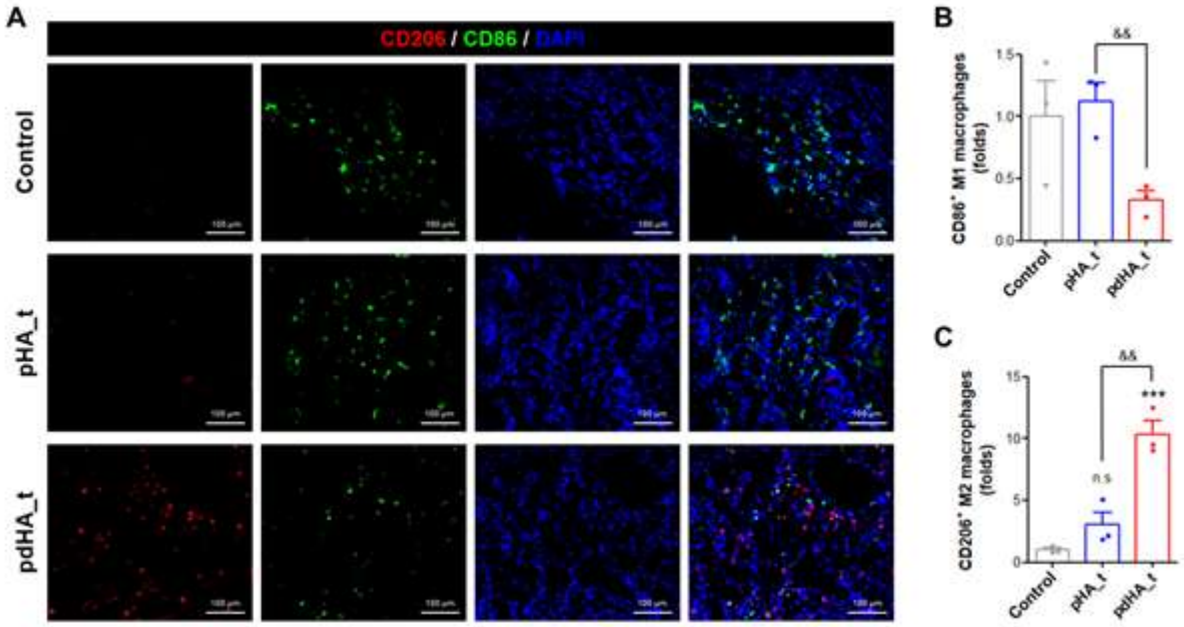

Figure S12. Comparison of distribution of $CD86^+$ M1 macrophages and $CD206^+$ M2 macrophages in the infarct area. A) Co-staining of CD206 and CD86 in the infarct area on day 28 in the Control, pHA_t, and pdHA_t groups. Scale bar = 100 μm. B) Comparison of distribution of $CD86^+$ M1 macrophages between groups (n=3). C) Comparison of distribution of $CD206^+$ M2 macrophages between groups (n=3). Data are presented as mean ± SEM. (*** < 0.001 (comparison with control); ns indicates no significance, and && < 0.01).
